# Effects of Psyllium Husk on Metabolic Regulators, Insulin Resistance, and SIRT6 in Liver and Muscle of Type 2 Diabetic Rats

**DOI:** 10.1002/vms3.70942

**Published:** 2026-04-06

**Authors:** Furkan Ümit, Gülay Çiftci, Burcu Onuk, Recai Aci, Özüm Çaka, Alper Çiftci

**Affiliations:** ^1^ Department of Veterinary Biochemistry Faculty of Veterinary Medicine Ondokuz Mayıs University Samsun Turkey; ^2^ Department of Veterinary Anatomy Faculty of Veterinary Medicine Ondokuz Mayıs University Samsun Turkey; ^3^ Söke Vocational School of Health Services Aydιn Adnan Menderes University Aydιn Turkey; ^4^ Department of Veterinary Microbiology Faculty of Veterinary Medicine University of Ondokuz Mayıs Samsun Turkey

**Keywords:** insulin resistance, oxidative stress, psyllium husk powder, type 2 diabetes mellitus

## Abstract

**Background:**

Type 2 diabetes mellitus (T2DM) is a common metabolic disorder marked by insulin resistance, impaired insulin secretion, oxidative stress, and dysregulated appetite and energy balance. Biomarkers like glucose transporter type 4 (GLUT4), phosphoinositide 3‐kinase (PI3K), sirtuin 6 (SIRT6), nesfatin‐1, glucagon‐like peptide 1 (GLP‐1), leptin, and insulin‐like growth factor 1 (IGF‐1) play important roles in various physiological processes.

**Objectives:**

This study evaluated the metabolic and molecular effects of psyllium in an experimental T2DM rat model.

**Methods:**

Thirty male Wistar Albino rats were divided into three groups (*n* = 10): Control, Diabetes, and Diabetes + PHP. T2DM was induced by a high‐fat diet followed by streptozotocin (35 mg/kg), and the Diabetes + PHP group received 10% PHP from week five. At study completion, metabolic parameters, serum biomarkers, and tissue protein expressions were assessed.

**Results:**

T2DM is typically associated with elevated insulin levels and overeating behaviour due to insulin resistance. However, PHP treatment appears to improve insulin sensitivity, leading to a reduction in both body weight and insulin levels. In the T2DM rat model, PHP significantly reduced serum glucose, triglyceride, and leptin levels compared with the diabetic group (*p* < 0.05). PHP reduced body weight and serum Homeostatic Model Assessment of Insulin Resistance (HOMA‐IR), insulin, total cholesterol, creatinine, C‐peptide, and total oxidant status, while increasing serum SIRT6, total antioxidant status, Homeostatic Model Assessment of β‐cell function (HOMA‐β), nesfatin‐1, glucagon‐like peptide‐1 (GLP‐1), and insulin‐like growth factor‐1 (IGF‐1); however, these changes were not statistically significant (*p* > 0.05). Tissue analyses showed that PHP improved some muscle parameters (triglycerides and total protein; *p* < 0.05) but did not fully normalise glucose levels in liver and muscle. Uric acid levels remained decreased in liver and muscle after PHP treatment. PHP tended to increase tissue insulin levels while reducing serum insulin, and partially modulated SIRT6 levels, without statistical significance. Sodium dodecyl sulfate‐polyacrylamide gel electrophoresis (SDS‐PAGE) revealed distinct group‐specific protein expression patterns in liver and muscle tissues. Additionally, GLUT4, PI3K, and SIRT6 expressions were reduced in diabetic rats and partially restored by PHP without reaching control levels.

**Conclusions:**

PHP demonstrated significant beneficial effects by partially improving metabolic dysfunctions and increasing antioxidant capacity in T2DM. These findings suggest that PHP may be a promising adjunctive therapeutic strategy for managing T2DM‐related abnormalities, potentially improving both insulin sensitivity and overall metabolic balance.

## Graphical abstract

The effects of psyllium husk supplementation on metabolic regulators, insulin resistance markers, and SIRT6 expression in the liver and skeletal muscle tissues of type 2 diabetic rats were investigated. PHP demonstration has significant beneficial effects by partially improving metabolic dysfunctions and increasing antioxidant capacity in T2DM.

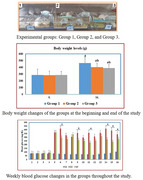

## 1 Introduction

Type 2 diabetes mellitus (T2DM) is a prevalent metabolic disorder characterised by chronic hyperglycaemia resulting from insulin resistance and impaired insulin secretion (American Diabetes Association 2023). It poses a significant global health burden due to its associated complications, including cardiovascular diseases, neuropathy, and nephropathy (Magliano et al. 2022). Insulin resistance, particularly in liver and skeletal muscle tissues, plays a central role in the pathophysiology of T2DM (DeFronzo and Tripathy 2009). Recent studies highlight the importance of metabolic regulators such as like glucose transporter type 4 (GLUT4) and phosphoinositide 3‐kinase (PI3K) in glucose uptake and insulin signalling pathways (Taniguchi et al. 2006). Additionally, sirtuin‐6 (SIRT6), a member of the sirtuin family, has emerged as a key regulator of glucose metabolism and inflammation, with protective effects against insulin resistance and metabolic dysfunction (Feldman et al. 2013).

Psyllium husk, a soluble dietary fibre derived from *Plantago ovata*, has demonstrated beneficial effects on glycaemic control, lipid profile, and insulin sensitivity in diabetic models and human studies (Pal et al. 2011; Vuksan et al. 2000). Its ability to modulate gut microbiota and reduce oxidative stress further supports its therapeutic potential in managing T2DM (Soliman 2019).

Despite these promising findings, the molecular mechanisms underlying the effects of psyllium husk on insulin resistance and key metabolic regulators such as SIRT6 have not yet been adequately investigated. Therefore, this study aimed to investigate the effects of psyllium husk supplementation on metabolic regulators, insulin resistance markers, and SIRT6 expression in the liver and skeletal muscle tissues of type 2 diabetic rats.

## 2 Materials and Methods

### 2.1 Ethics Approval

The study was approved by the Ondokuz Mayıs University Animal Experiments Local Ethics Committee (HAYDEK) under decision number 2022/58. Experimental procedures were carried out at the Ondokuz Mayıs University Experimental Animals Research and Application Center (DEHAM).

### 2.2 Diets (ST, HFD, HFD + PHP Supplemented Diet)

**Control diet (ST)**: The control diet consisted of 24% protein, 4% fat, and 34.39% carbohydrates, with 4.68% sugar, 29.71% starch, and 6.28% crude fibre. Additionally, it contained 6.06% vitamins and minerals, providing approximately 2.716 kcal/g of energy. This diet was based on standard.

**High‐fat diet (HFD)**: The HFD contained 18.30% protein, 21.70% carbohydrates, and 60% fat, providing 4.057 kcal/g. Its composition included 730.64 g of casein, 12 g of L‐cysteine, 500 g of maltodextrin, 344.36 g of sucrose, 225 g of corn oil, 2205 g of palm oil, and 40 g of vitamin mix (V10001) (D12492, ARDEN, Ankara).

**FIGURE 1:**
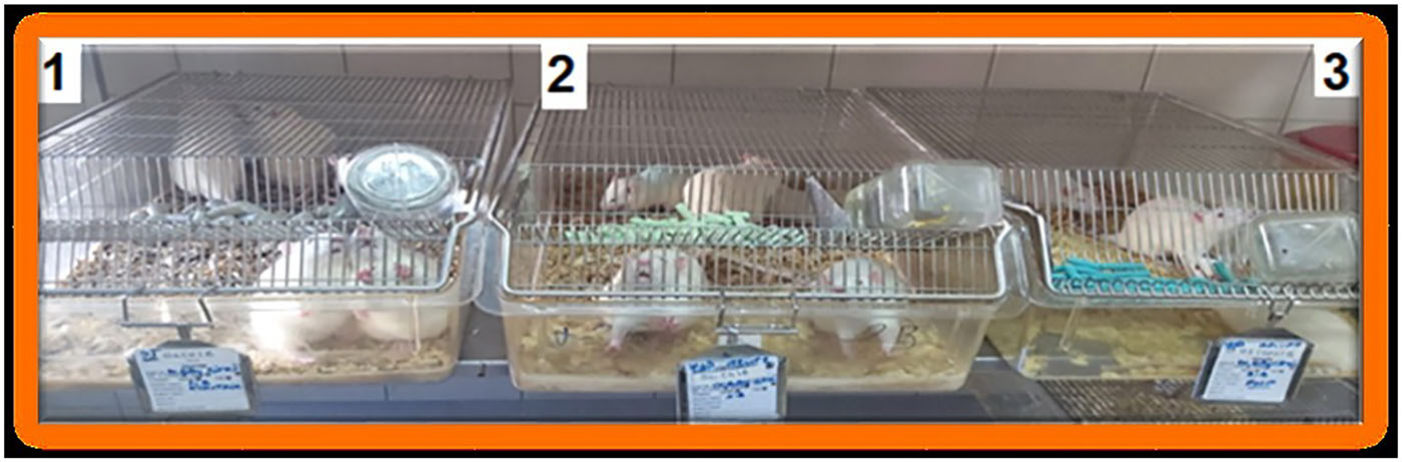
Experimental groups: Group 1 (1), Group 2 (2), and Group 3 (3).

**High‐fat diet + 10% psyllium husk powder (HFD + 10% PHP)**: The HFD + 10% PHP had the same macronutrient composition but slightly lower energy (4.057 kcal). The only change was the replacement of 344.36 g of sucrose with 311 g of psyllium husk powder, while the other ingredients remained unchanged (D12492, ARDEN, Ankara) (Ogata et al. 2017). Rats were given ad libitum access to both their respective diets and water throughout the study (Figure 1). Additionally, an acclimatisation period was provided before feeding the experimental high‐fat diet, during which the animals were allowed to adjust to the housing and feeding conditions.

### 2.3 Formation of Experimental Groups

Environmental conditions were strictly controlled with a 12/12‐h light/dark cycle (lights on at 7:30, off at 19:30), temperature maintained at 22 ± 2°C, and humidity at 55 ± 5%. The sample size was determined based on a prior power analysis, considering 80% power and a significance level (α) of 0.05. A total of 30 male Wistar Albino rats were used: 20 were induced with experimental T2DM, while 10 fed standard chow served as healthy controls.

Diabetes was induced in overnight fasted rats with a single intraperitoneal injection of streptozotocin (STZ) dissolved in cold 0.1 M sodium citrate buffer (pH 4.5) at a dose of 35 mg/kg body weight. For the control groups, 10 rats were injected intraperitoneally with 0.1 M sodium citrate buffer as the vehicle. Following the injections, the animals were given a 5% dextrose solution overnight to prevent hypoglycaemic shock, and they had free access to a high‐fat diet. Three days after STZ injection, fasting blood glucose levels were measured from the tail vein using a glucometer (Accu‐Check Instant, Roche ve Optima, OkMeter) to confirm successful induction of diabetes. Subsequently, glucose levels were monitored weekly (Ermiş and Çiftci 2024). Rats with fasting blood glucose levels above 200 mg/dL were classified as diabetic. The normal glucose range for the healthy control group was defined as 90–110 mg/dL.

**Group 1 (Control, G1, *n* = 10)**: Rats were fed standard chow and given water ad libitum for 16 weeks. At week 5, animals received intraperitoneal injections of 0.1 M citrate phosphate buffer. On all other days, 100 µL of saline was administered intraperitoneally.

**Group 2 (Diabetes, G2, *n* = 10)**: Rats were fed a high‐fat diet (HFD) for 4 weeks, followed by a single intraperitoneal injection of STZ (35 mg/kg) dissolved in 0.1 M citrate phosphate buffer to induce diabetes. After STZ administration, rats continued on the HFD for the remainder of the study (Furman 2021).

**Group 3 (Diabetes + Psyllium Husk Powder, G3, *n* = 10)**: Rats were fed HFD for 4 weeks, with diabetes induced as in Group 2. After STZ injection, rats were maintained on HFD supplemented with 10% psyllium husk powder (PHP) for 12 weeks (Ogata et al. 2017).

At the end of the 16‐week period, rats were fasted for 12 h with access to water before being weighed individually. Euthanasia was performed under general anaesthesia using sodium pentobarbital (30 mg/kg, intraperitoneal) and ketamine hydrochloride (10 mg/kg, intramuscular). Following anaesthesia, cardiac blood was collected, and necropsies were conducted. Liver and skeletal muscle tissues were harvested; muscle samples were taken from the right hind limb.

Blood samples were allowed to clot at room temperature for 20 min, then centrifuged at 1550 × *g* for 10 min at +4°C. Serum was separated, aliquoted, and stored at −20°C until further biochemical analysis.

### 2.4 Determination of Body Weight Change

Individual body weights were measured at the beginning and end of the experiment to calculate body weight gain.

### 2.5 Evaluation of Glucose Homeostasis

Glucose homeostasis was evaluated by assessing insulin resistance and pancreatic β‐cell function. Insulin resistance was determined using the Homeostasis Model Assessment of Insulin Resistance (HOMA‐IR), while β‐cell function was evaluated using the β‐cell function index (HOMA‐β), calculated according to the following formulas (Bonora et al. 2000).

$$\mathrm{HOMA}-\mathrm{IR}=\left(\mathrm{Fasting}\mathrm{Blood}\mathrm{Glucose}\times \mathrm{Fasting}\mathrm{Insulin}\right)/405,$$

HOMA−β(%)=[Insulin(μIU/mL)×360]/[(Glucose(mg/dL)−63)].

### 2.6 Biochemical Analysis

Levels of glucose (lot: 11803), creatinine (lot: 11802), total cholesterol (lot: 11805), triglycerides (lot: 11828), total protein (lot: 11800), urea (lot: 11516), albumin (lot: 11573), and uric acid (lot: 11821) in serum, liver, and muscle tissues were measured using specific Biosistem kits and analysed by spectrophotometry on an automated analyser (Biosistem A25, Spain).

### 2.7 Oxidative Stress Parameters

Serum TAS was measured using the Rel Assay Diagnostics Kit (Mega Tıp, Gaziantep, Turkey) via a colorimetric method based on hydroxyl radical production. This method utilises the ABTS radical, which loses its blue‐green colour proportionally to antioxidant concentration. Absorbance changes were measured at 660 nm. The assay relies on the oxidation of ABTS to ABTS^+^ in the presence of hydrogen peroxide. The reaction rate was calibrated using Trolox, and results were expressed as mmol Trolox equivalent/L (Erel 2004).

TOS was measured using the Rel Assay (Rel Assay Diagnostics kit, Mega Tıp, Gaziantep, Turkey) with a colorimetric method developed by Erel (2005). Oxidants in the sample oxidise ferrous ion‐o‐dianisidine to ferric ions, and glycerol accelerates this reaction. Ferric ions form a coloured complex with xylenol orange in an acidic environment. The intensity of the colour, related to the amount of oxidants, was measured spectrophotometrically and calibrated with hydrogen peroxide. Results were expressed in µmol H_2_O_2_ equivalent/L (Erel 2005).

The Oxidative Stress Index (OSI) was calculated by converting TAS results to mmol/L and using the following formula (Giorgio and Quattrocchi 2024). OSI (arbitrary unit) = TOS (µmol H_2_O_2_ equivalent/L) / TAS (µmol Trolox equivalent/L) × 10^−^^1^.

### 2.8 Serum Hormone and Protein Measurements

Serum levels of Nesfatin‐1 (BT LAB, ELISA kit, cat. no. E0878Ra), insulin (cat. no. E0707Ra), GLP‐1 (cat. no. E0709Ra), Insulin‐like Growth Factor‐1 (IGF‐1) (cat. no. E0709Ra), Sirtuin‐6 (cat. no. E1520Ra), C‐peptide (cat. no. E0006Ra), and leptin (cat. no. E0561Ra) were measured using rat‐specific ELISA kits (BT LAB). Each assay was performed according to the manufacturer's instructions, and optical density (OD) at 450 nm was analysed with Magellan Standard Tracker software (V7–2) based on the standard curves.

### 2.9 Preparation of Liver and Muscle Tissue Homogenate

Liver and muscle tissue homogenisation was performed according to the ELISA test kit procedure. First, the tissue was washed with ice‐cold PBS (0.01 mol/L, pH 7.2–7.4) and weighed. Tissues were washed in ice‐cold PBS (pH 7.4) to thoroughly remove excess blood and weighed before homogenisation. Tissues were minced and homogenised in PBS (tissue weight (g): PBS (mL) volume = 1:9) with a homogeniser on ice. To further disrupt cells, the suspension could be sonicated with an ultrasonic cell disruptor or subjected to freeze‐thaw cycles. Homogenates were then centrifuged at 5000 × *g* for 5 min to obtain the supernatant. The supernatant was separated into aliquots and stored at −20°C until analysis.

### 2.10 Determination of SIRT6 and Insulin Levels in Liver and Muscle Tissue

Levels of SIRT6 and insulin in liver and muscle tissue supernatants were measured using rat‐specific ELISA kits (BT LAB; Sirtuin‐6: Cat. No. E1520Ra, Insulin: Cat. No. E0707Ra), according to the manufacturers’ instructions.

### 2.11 Determination of Protein Levels in Liver and Muscle Tissues

Protein quantification was performed using the BCA protein assay kit (Thermo Fisher Scientific, cat. no. 23225) with bovine serum albumin (BSA) as the standard. BSA standards ranging from 0 to 2000 µg/mL were prepared and loaded into a 96‐well plate. Protein samples were diluted 1:50, and 20 µL of each sample, standard, and blank were pipetted in duplicate. A mixture of Solutions A and B was added to each well, followed by incubation at 37°C for 30 min. Absorbance was measured at 592 nm, and protein concentrations were used to normalise sample amounts for subsequent analyses.

### 2.12 Protein Profiling and Western Blot Analysis

To determine the protein profile in liver and muscle tissues, 40 µg of protein from tissue lysates was loaded into each well for SDS‐PAGE analysis (Laemmli 1970). Lysates were mixed 1:1 with sample buffer (0.0625 M Tris‐HCl, pH 6.8; 2% SDS; 5% 2‐mercaptoethanol; 10% glycerol; 0.02% bromophenol blue), then boiled at 100°C for 5 min. SDS‐PAGE was performed under reducing conditions using 10% polyacrylamide gels in a Bio‐Rad Mini‐PROTEAN Tetra Vertical Electrophoresis system. Protein separation was carried out in a Tris‐glycine buffer at a constant current of 40 mA (20 mA per gel).

Following electrophoresis, the gels were stained with Coomassie Brilliant Blue G‐250 to visualise the protein profile (Candiano et al. 2004). For specific protein analysis of GLUT4, SIRT6, and PI3K, proteins were transferred onto polyvinylidene fluoride (PVDF) membranes using a semi‐dry blotting apparatus at 15 V for 30 min. The transfer buffer consisted of 2.5 mM Tris (pH 8.3), 0.02 M glycine, and 20% methanol (v/v) (Burnette 1981).

Membranes were blocked with a solution containing 0.1% Tween‐20 in PBS (pH 7.4) for 1 h at room temperature. Following blocking, membranes were incubated overnight at 4°C on an orbital shaker with primary antibodies diluted in the blocking solution: GLUT4 (Cloud‐Clone Corp, PACO23Ra01), PI3K (Cloud‐Clone Corp, PAJ826Ra01), and SIRT6 (Cloud‐Clone Corp, PAE916Ra01). Following primary antibody incubation, membranes were washed with Tris‐buffered saline (TBS) with 0.1% (v/v) Tween‐20 (TBS‐T) and then incubated for 1 h with an HRP‐conjugated goat anti‐rat IgG secondary antibody, diluted in blocking solution. After additional washes with TBS‐T, protein bands were visualised using 0.6 mg/mL 3,3'‐diaminobenzidine (DAB; Sigma) and 0.12% H_2_O_2_ in 1 M Tris‐HCl. The resulting band intensities were quantified and compared to the control group using commercial image analysis software.

### 2.13 Statistical Analysis

Statistical analyses were performed using SPSS version 22.0 (IBM Corp., Armonk, NY, USA). The normality of the data was assessed using the Shapiro–Wilk test. For normally distributed data, group comparisons were performed using one‐way analysis of variance (ANOVA), followed by Duncan's multiple range test for post hoc analysis. For non‐normally distributed data, the Kruskal–Wallis test was applied. Pearson correlation analysis was used for parametric data, while Spearman correlation was used for non‐parametric data. Results are presented as mean ± standard error of the mean (SEM). A *p*‐value of less than 0.05 was considered statistically significant.

## 3 Results

### 3.1 Body Weight Change of the Groups

**FIGURE 2:**
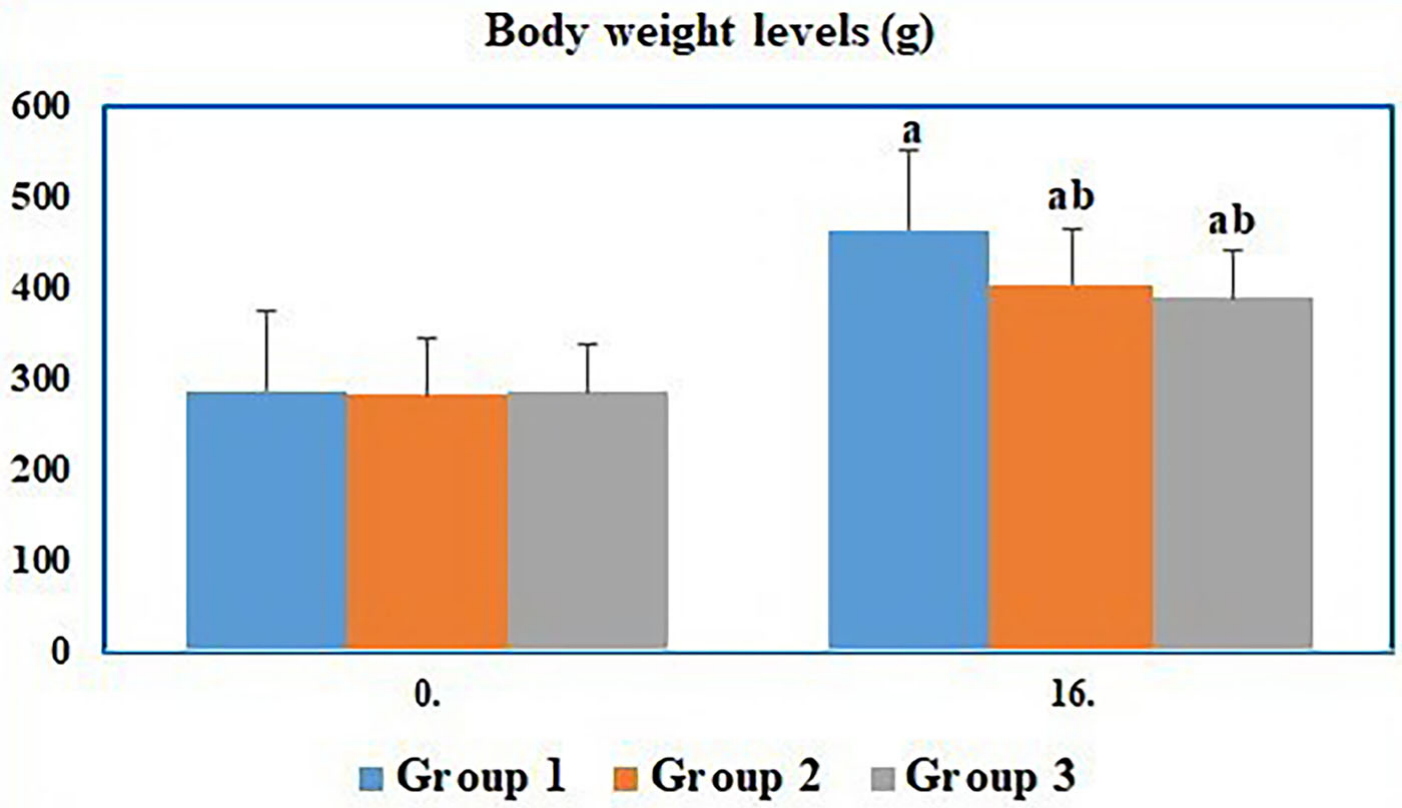
Body weight changes of the groups at the beginning and end of the study.

At the beginning of the study (week 0), body weights were similar across all groups (Group 1, Group 2, and Group 3), with no statistically significant differences observed (*p* > 0.05). By the end of the experiment (week 16), Group 1 exhibited the greatest body weight gain, while body weights decreased in the diabetic groups compared to the control. Although psyllium husk powder supplementation in Group 3 slightly reduced body weight compared to Group 2, the difference was not statistically significant (*p* > 0.05) (Table 1 and Figure 2).

**TABLE 1:** Body weight level (g) changes of rats.

Week	Group 1	Group 2	Group 3
**0**	287.3	281.49	284.8
**16**	462.5^a^	403.4^ab^	387.6^ab^

^a,b^Differences between groups indicated by different letters in the same row are significant (*p* < 0.05).

### 3.2 Evaluation of Glucose Homeostasis

At the beginning (weeks 1–4), all groups (G1, G2, G3) had similar blood glucose levels, with Group 1 fed standard chow showing slightly lower glucose levels compared to Groups 2 and 3 fed a HDF (*p* < 0.05).

**FIGURE 3:**
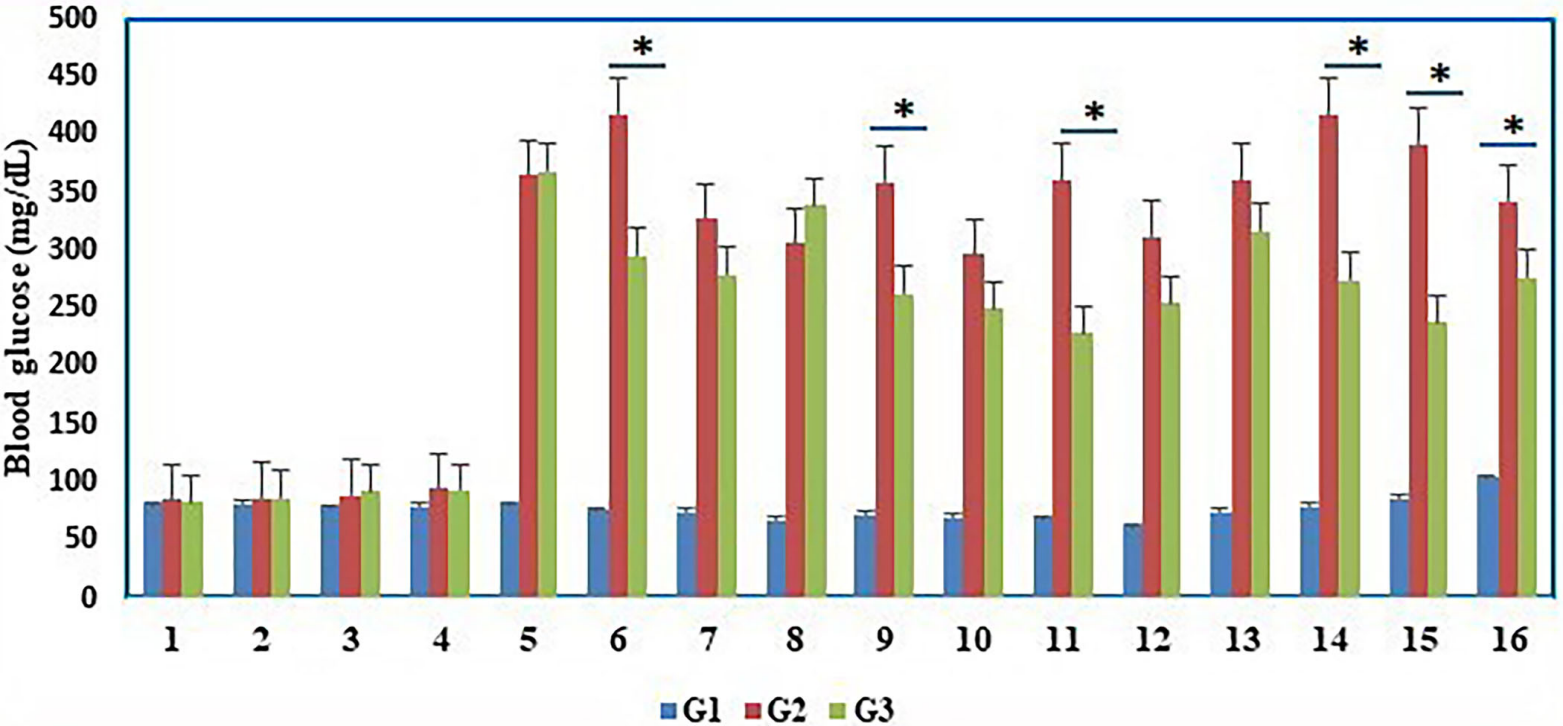
Weekly blood glucose changes in the groups throughout the study.

At week 5, following STZ injection to induce diabetes in Groups 2 and 3, blood glucose levels in these groups significantly increased (∼365 mg/dL) (*p* < 0.05), while Group 1 remained stable at approximately 90 mg/dL. Between weeks 6 and 16, Groups 2 and 3 exhibited sustained hyperglycaemia (>200 mg/dL), confirming diabetic status. Notably, Group 3, supplemented with PHP, demonstrated significantly lower blood glucose levels compared to Group 2 at multiple time points (weeks 6, 9, 11, 14, 15, and 16) (*p* < 0.05), suggesting that psyllium husk supplementation partially mitigated hyperglycaemia (Table 2 and Figure 3).

**TABLE 2:** Weekly blood glucose levels (mg/dL) of rats.

Weeks	G1	G2	G3
**1**	80.6 ± 1.8	85.1 ± 3	82.5 ± 2.1
**2**	82 ± 2.4	86.7 ± 2.9	86.4 ± 1.6
**3**	78.1 ± 2.8^a^	88.6 ± 2.5^b^	92.3 ± 3.8^b^
**4**	79.2 ± 2^a^	94.3 ± 3.3^b^	92.8 ± 3.7^b^
**5**	80.4 ± 2.3^a^	364.6 ± 21.4^b^	368.6 ± 17.4^b^
**6**	75.3 ± 2.9^a^	417.1 ± 34^b^	295.3 ± 45^c^
**7**	74.9 ± 3.7^a^	327.3 ± 24.3^b^	279.1 ± 32.1^b^
**8**	67.5 ± 2.7^a^	305.7 ± 32.9^b^	339.4 ± 37.4^b^
**9**	72.3 ± 2.7^a^	359 ± 37.1 ^b^	263.1 ± 32 ^c^
**10**	70.3 ± 2.1^a^	296.1 ± 16.6 ^b^	249.2 ± 28 ^b^
**11**	68.9 ± 2.4 ^a^	360.4 ± 34.3 ^b^	227.9 ± 23.2 ^c^
**12**	61.5 ± 1.8 ^a^	311.7 ± 30.7 ^b^	254.5 ± 27.5 ^b^
**13**	75.1 ± 4.4 ^a^	360.6 ± 39.4 ^b^	317.1 ± 47.6 ^b^
**14**	79.2 ± 4.3 ^a^	416.6 ± 33.3 ^b^	274.3 ± 42.5 ^c^
**15**	86.1 ± 3.1 ^a^	391.4 ± 23.6 ^b^	237.4 ± 21.3 ^c^
**16**	104.42 ± 3.6 ^a^	370.11 ± 27.74 ^b^	276.3 ± 43.2 ^c^

^a,b,c^Differences between groups marked with different letters in the same row are significant (*p* < 0.05).

In our study, diabetes induced by HFD and STZ caused significant alterations in insulin, glucose, HOMA‐IR, and HOMA‐β parameters (*p* < 0.05). Diabetic groups (G2 and G3) showed significantly higher insulin levels than the control group (G1), although insulin levels in G3 did not differ significantly from G1. Blood glucose, HOMA‐IR, and HOMA‐β (%) were significantly elevated in G2 and G3 compared to controls, indicating insulin resistance and β‐cell dysfunction (*p* < 0.05). Psyllium husk supplementation (G3) significantly improved these parameters relative to the untreated diabetic group (G2) (*p* < 0.05), but values remained significantly different from controls (*p* < 0.05). The results are presented in Table 3.

**TABLE 3:** Insulin, glucose, HOMA‐IR, and HOMA‐β levels.

	G1	G2	G3
**Insulin (mlU/L)**	5.86 ± 1.18^a^	6.25 ± 0.^7b^	5.68 ± 0.7^b^
**Glucose (mg/dL)**	115.08 ± 7.27	376.22 ± 24.82^b^	219.87 ± 17.4^c^
**HOMA‐IR**	1.7 ± 0.1^a^	5.32 ± 0.7^b^	3.96 ± 0.7^b^
**HOMA‐β (%)**	53.48 ± 4.7^a^	10.05 ± 1.7^b^	14.04 ± 3.5^b^

^a,b^Differences between groups indicated by different letters in the same row are significant (*p* < 0.05).

It was determined that there is a significant positive correlation between glucose levels and HOMA‐IR (*r* = 0.974**), a significant negative correlation between HOMA‐IR and HOMA‐β (*r* = −0.674**), and a significant positive correlation between HOMA‐IR and insulin levels (*r* = 0.432*) (Table 4).

**TABLE 4:** Correlation relationships of serum glucose, HOMA‐IR, HOMA‐β, and insulin levels.

	Glucose	HOMA‐IR	HOMA‐β	Insulin
**Glucose**	1	0.974^**^	0.159	0.283
**HOMA‐IR**		1	−0.674^**^	0.432^*^
**HOMA‐β**			1	0.159
**Insulin**				1

*p < 0.05.

**p < 0.01.

### 3.3 Biochemical Parameters in Serum, Liver, and Muscle Tissues

At the end of the 16‐week trial, the serum levels of glucose (Glu), total cholesterol (TC), triglycerides (TG), total protein (TP), albumin (ALB), uric acid (UA), urea (U), and creatinine (CRE) were presented in Table 5.

**TABLE 5:** Serum levels of total cholesterol (TC), triglycerides (TG), total protein (TP), albumin (ALB), uric acid (UA), urea (U), and creatinine (CRE).

	G1	G2	G3
**TC (mg/dL)**	48.1 ± 4.8^a^	109.33 ± 23.4^b^	71.78 ± 3.1^ab^
**TG (mg/dL)**	55.23 ± 2.4^a^	149.83 ± 24.7^b^	85.49 ± 4.9^a^
**TP (g/L)**	64.4 ± 1.5	60.88 ± 1.5	61.5 ± 0.8
**ALB (g/L)**	30.28 ± 1.3	30.65 ± 1	30.55 ± 0.2
**UA (mg/dL)**	1.27 ± 0.09	1.61 ± 0.1	1.5 ± 0.1
**CRE (mg/dL)**	0.25 ± 0.01^a^	0.31 ± 0.02^b^	0.28 ± 0.01^ab^
**U (mg/dL)**	40.26 ± 1.4	42.22 ± 2.5	41.91 ± 1.3

^a,b^ Differences between groups indicated by different letters in the same row are significant (*p* < 0.05).

In the comparison of serum biochemical parameters, statistically significant differences were observed among the groups in terms of TC and TG levels (*p*  < 0.05). These differences indicated that a marked increase occurred in the diabetes group, while the values in the group treated with PHP were found to approach those of the control group. Significant differences were also found in CRE levels among the groups (*p*  < 0.05); an increase was observed in the diabetes group, and this increase was determined to be partially reduced with psyllium supplementation. On the other hand, no statistically significant differences were detected among the groups regarding TP, ALB, UA, and U levels (*p*  > 0.05). These findings showed that PHP may have beneficial effects on certain biochemical parameters.

The biochemical parameter levels in liver (urea; KC‐U, triglycerides; KC‐TG, total protein; KC‐TP, total cholesterol; KC‐TC, glucose; KC‐Glu, uric acid; KC‐UA) and muscle (urea; K‐U, triglycerides; K‐TG, total protein; K‐TP, total cholesterol; K‐TC, glucose; K‐Glu, uric acid; K‐UA) tissues are presented in Table 6.

**TABLE 6:** Levels of biochemical parameters in liver (KC) and muscle (K) tissues.

	Group 1	Group 2	Group 3
**K‐U (mg/g)**	5.3 ± 0.38^a^	7.25 ± 0.61^b^	7.15 ± 0.76^ab^
**K‐TG (mg/g)**	21.87 ± 3.36^a^	35.33 ± 4.28^b^	24.04 ± 2.71^a^
**K‐TP (g/g)**	4 ± 0.21^ab^	3.66 ± 0.16^a^	4.5 ± 0.3^b^
**K‐TC (mg/g)**	1.41 ± 0.26	1.36 ± 0.26	1.16 ± 0.18
**K‐Glu (mg/g)**	4.88 ± 0.3^a^	9.46 ± 1.08^b^	8.22 ± 0.5^b^
**K‐UA (mg/g)**	0.56 ± 0.06^a^	0.37 ± 0.05^b^	0.24 ± 0.03^b^
**KC‐U (mg/g)**	9.58 ± 0.5	8 ± 0.7	8.33 ± 0.5
**KC‐TG (mg/g)**	142.49 ± 5.9^a^	114.96 ± 9.4^b^	121.54 ± 7.6^ab^
**KC‐TP (g/g)**	11 ± 1.36	9.77 ± 0.61	11.7 ± 0.77
**KC‐TC (mg/g)**	9.65 ± 1.26	9.73 ± 1.06	11.84 ± 1.09
**KC‐Glu (mg/g)**	78.91 ± 0.1^a^	187.86 ± 0.1^b^	184.37 ± 0.3^b^
**KC‐UA (mg/g)**	16.32 ± 0.4^a^	13.2 ± 1.2^ab^	12.52 ± 1.59^b^

^a,b^Differences between groups marked with different letters in the same row are significant (*p* < 0.05).

Abbreviations: KC‐U, liver urea; KC‐TG, liver triglycerides; KC‐TP, liver total protein; KC‐TC, liver total cholesterol; KC‐Glu, liver glucose; KC‐UA, liver uric acid;K‐U, muscle urea; K‐TG, muscle triglycerides; K‐TP, muscle total protein; K‐TC, muscle total cholesterol; K‐Glu, muscle glucose; K‐UA, muscle uric acid.

In our study, significant effects of diabetes on biochemical parameters in muscle and liver tissues were observed. In muscle tissue, urea (K‐U, *p* < 0.05) and triglyceride (K‐TG, *p* < 0.05) levels in the diabetic group (Group 2) were significantly increased compared to the control group (Group 1), while the group supplemented with psyllium (Group 3) showed levels approaching those of the control group. Total protein (K‐TP) levels were significantly increased (*p* < 0.05) in Group 3 receiving psyllium supplementation compared to the control group, whereas a marked decrease was observed in the diabetic group. Glucose (K‐Glu) levels were significantly elevated (*p* < 0.05) in both diabetic and supplemented groups compared to the control group; however, this increase was reduced in the psyllium group compared to the diabetic group. Uric acid (K‐UA) levels were significantly decreased (*p* < 0.05) in both diabetic and supplemented groups.

In liver tissue, triglyceride (KC‐TG, *p* < 0.05) and glucose (KC‐Glu, *p* < 0.05) levels were significantly increased in the diabetic group, whereas a reduction was observed with psyllium supplementation. Uric acid (KC‐UA) levels were significantly decreased (*p* < 0.05) in diabetic groups compared to controls. No significant differences were observed among groups for other parameters (KC‐URE, KC‐TP, KC‐TK) (*p* > 0.05) (Table 6).

These findings indicate that diabetes caused significant metabolic alterations in muscle and liver tissues, and PHP provided significant improvements in some metabolic parameters. However, the lack of statistical significance in certain parameters (*p* > 0.05) suggests that the benefits of psyllium supplementation may be limited.

It was determined that there was a positive correlation between KC‐URE and KC‐TG (*r* = 0.560**) and between KC‐URE and KC‐TP (*r* = 0.407*), while there was a negative correlation between K‐TP and KC‐TG (*r* = −0.372*). A positive correlation was observed between KC‐TK and KC‐TP (*r* = 0.897**), as well as between KC‐Glu and K‐TG (*r* = 0.530**). Additionally, there was a positive correlation between K‐URE and K‐Glu (*r* = 0.586**), and between K‐TG and both K‐Glu and KC‐Glu (*r* = 0.516**, *r* = 0.530**), and between K‐Glu and KC‐Glu (*r* = 0.677**).

### 3.4 Oxidative Stress Parameters

The levels of TAS, TOS, and OSI in serum are presented in Tables 7 and 8, along with the corresponding figures.

**TABLE 7:** Serum TAS, TOS, and OSI levels.

	G1	G2	G3
**TAS (mmol/L)**	1.87 ± 0.06^ab^	1.51 ± 0.1^a^	1.82 ± 0.2^ab^
**TOS (µmol/L)**	4.29 ± 1.1^a^	15.58 ± 1.8^b^	15.12 ± 1.7^b^
**OSI (arbitrary units)**	0.22 ± 0.06^a^	1.08 ± 0.1^b^	0.87 ± 0.1^b^

^a,b,c^ Differences between groups indicated with different letters in the same row are significant (*p* < 0.05). Values ​​represent the mean ± standard error of TOS total oxidant status, TAS total antioxidant status, OSI oxidative stress index.

**TABLE 8:** Correlation relationship of TAS, TOS, and OSI levels.

	TAS	TOS	OSI
**TAS**	1	−0.196	0.893^*^
**TOS**		1	−0.548^*^
**OSI**			1

*p < 0.01.

### 3.5 Serum Hormone and Protein Measurements

The serum levels of nesfatin‐1, insulin, GLP‐1, leptin, IGF‐1, C‐peptide, and SIRT6 in the groups are presented in Tables 9 and 10.

**TABLE 9:** The average serum levels of nesfatin‐1, insulin, GLP‐1, leptin, insulin‐like growth factor (IGF‐1), C‐peptide, and sirtuin‐6.

	G1	G2	G3
**Nesfatin‐1 (ng/L)**	238. ± 11.8	226.25 ± 16.11	252.46 ± 3.5
**GLP‐1 (ng/L)**	232.11 ± 25.3	203.85 ± 13.9	216.45 ± 9.1
**IGF‐1 (ng/mL)**	102.62 ± 7.2	89.81 ± 9.7	96.58 ± 3.8
**Leptin (ng/mL)**	243.31 ± 12.9^a^	277.96 ± 7.3^b^	250.9 ± 5.6^a^
**C‐peptide (ng/mL)**	8.51 ± 0.2	8.73 ± 0.4	8.45 ± 0.1
**Insulin (mlU/L)**	5.79 ± 0.4	6.16 ± 0.23	5.86 ± 0.24
**Sirtuin‐6 (ng/mL)**	5.56 ± 0.5^a^	4.42 ± 0.1^b^	4.61 ± 0.1^b^

^a,b^ Differences between groups marked with different letters in the same row are significant (*p* < 0.05).

**TABLE 10:** Correlation relationships of biochemical parameters in the serum.

	Glu	TC	TG	TP	ALB	UA	CRE	U
**Glu**	1	0.543^**^	0.811^**^	−0.459^*^	−0.061	0.342	0.370^*^	0.344
**TC**		1	0.773^**^	−0.067	0.380^*^	0.773^**^	0.479^**^	−0.06
**TG**			1	−0.242	0.186	0.755^**^	0.388^*^	0.338
**TP**				1	0.423^*^	−0.267	−0.107	−0.325
**ALB**					1	0.110	−0.044	0.08
**UA**						1	0.464^*^	0.113
**CRE**							1	−0.351
**U**								1

Abbreviations: TC, total cholesterol; TG, triglycerides; TP, total protein; ALB, albumin; UA, uric acid; CRE, creatinine; U, urea.

*p < 0.05.

** *p* < 0.01.

Nesfatin‐1 levels were lowest in Group 2, and supplementation with PHP showed a positive effect by increasing these levels. However, the differences between the groups were not statistically significant (*p* > 0.05).

GLP‐1 and IGF levels were highest in the control group (G1) and decreased in the diabetic groups (G2 and G3) compared to the control. However, in the group supplemented with 10% PHP (G3), these levels increased, approaching those of the control group (*p* > 0.05). Leptin levels were lowest in the control group (G1), highest in the diabetic group (G2) with a statistically significant difference (*p* < 0.05), and decreased in the PHP group (G3), approaching control levels (*p* > 0.05).

The C‐peptide levels were highest in G2, followed by G1 and G3, with no statistically significant difference observed between the groups (*p* > 0.05). Insulin levels were also highest in Group 2, followed by G1 and G3, with no statistically significant difference between the groups (*p* > 0.05).

SIRT6 levels were highest in the control group (G1) and lowest in the diabetic group (G2) (*p* < 0.05). In the group with diabetes that was supplemented with 10% PHP (G3), sirtuin‐6 levels were found to be lower than in G1 but higher than in G2 (*p* > 0.05) (Table 11).

**TABLE 11:** Insulin and sirtuin‐6 protein levels in liver, muscle tissue, and serum.

	G1	G2	G3
**KC‐Insulin (mLU/g)**	2.66 ± 0.3	2.39 ± 0.2	3.14 ± 0.2
**KC‐Sirtuin‐6 (ng/mL)**	1.66 ± 0.2	1.84 ± 0.3	1.99 ± 0.9
**K‐Insulin (mLU/g)**	2.81 ± 0.3	2.41 ± 0.4	2.54 ± 0.2
**K‐Sirtuin‐6 (ng/g)**	2.28 ± 0.2^a^	2.12 ± 0.2^ab^	1.63 ± 0.1^a^
**S‐Insulin (mLU/L)**	5.86 ± 0.3	6.25 ± 0.23	5.68 ± 0.2
**S‐Sirtuin‐6 (ng/L)**	5.37 ± 0.4^a^	4.41 ± 0.1^b^	4.61 ± 0.1^ab^

^a,b^ Differences between groups indicated with different letters in the same row are significant (*p* < 0.05).

Abbreviations: KC‐Insulin, liver insulin; KC‐Sirtuin‐6, liver sirtuin‐6; K‐Insulin, muscle insulin; K‐Sirtuin‐6, muscle sirtuin‐6; S‐Insulin, serum insulin; S‐Sirtuin‐6, serum sirtuin‐6.

### 3.6 Insulin and SIRT6 Protein Levels in Liver, Muscle Tissue, and Serum

The insulin and sirtuin‐6 protein levels in the liver, muscle tissues, and serum are presented in Table 11.

It was determined that insulin levels were the lowest in liver and muscle tissue in Group 2 and the highest in serum in Group 2. In the group in which diabetes was induced and 10% PHP was given (G3), insulin levels in liver and muscle tissue increased compared to the diabetes group (G2) and decreased in serum, approaching the control group level (*p* > 0.05).

It was determined that sirtuin‐6 level increased in liver tissue in diabetic groups (G2 and G3) compared to the control group (G1) (*p* > 0.05) and decreased in muscle tissue and serum (*p* > 0.05). The correlation between liver (KC) and muscle (K) tissue values and serum (S) insulin and SIRT6 levels was investigated. Although both positive and negative correlations were observed between insulin and sirtuin‐6 levels, these correlations were not statistically significant (Table 12).

**TABLE 12:** Relationship between insulin and sirtuin‐6 levels of liver (KC), muscle tissue (K), and serum (S).

	KC‐Insulin	KC‐Sirtuin‐6	K‐Insulin	K‐Sirtuin‐6	S‐Insulin	S‐Sirtuin‐6
**KC‐ Insulin**	1	0.361	−0.054	−0.089	−0.151	0.158
**KC‐Sirtuin‐6**		1	0.141	−0.258	0.009	0.146
**K‐ Insulin**			1	0.143	0.076	0.156
**K‐Sirtuin‐6**				1	0.148	0.224
**S‐ Insulin**					1	0.207
**S‐Sirtuin‐6**						1

Abbreviations: KC‐Insulin, liver insulin; KC‐Sirtuin‐6, liver sirtuin‐6; K‐Insulin, muscle insulin; K‐Sirtuin‐6, muscle sirtuin‐6; S‐Insulin, serum insulin; S‐Sirtuin‐6, serum sirtuin‐6.

### 3.7 Protein Expression of GLUT4, PI3K, IRS‐1, and IRS‐2 in Liver and Muscle Tissues

**FIGURE 4:**
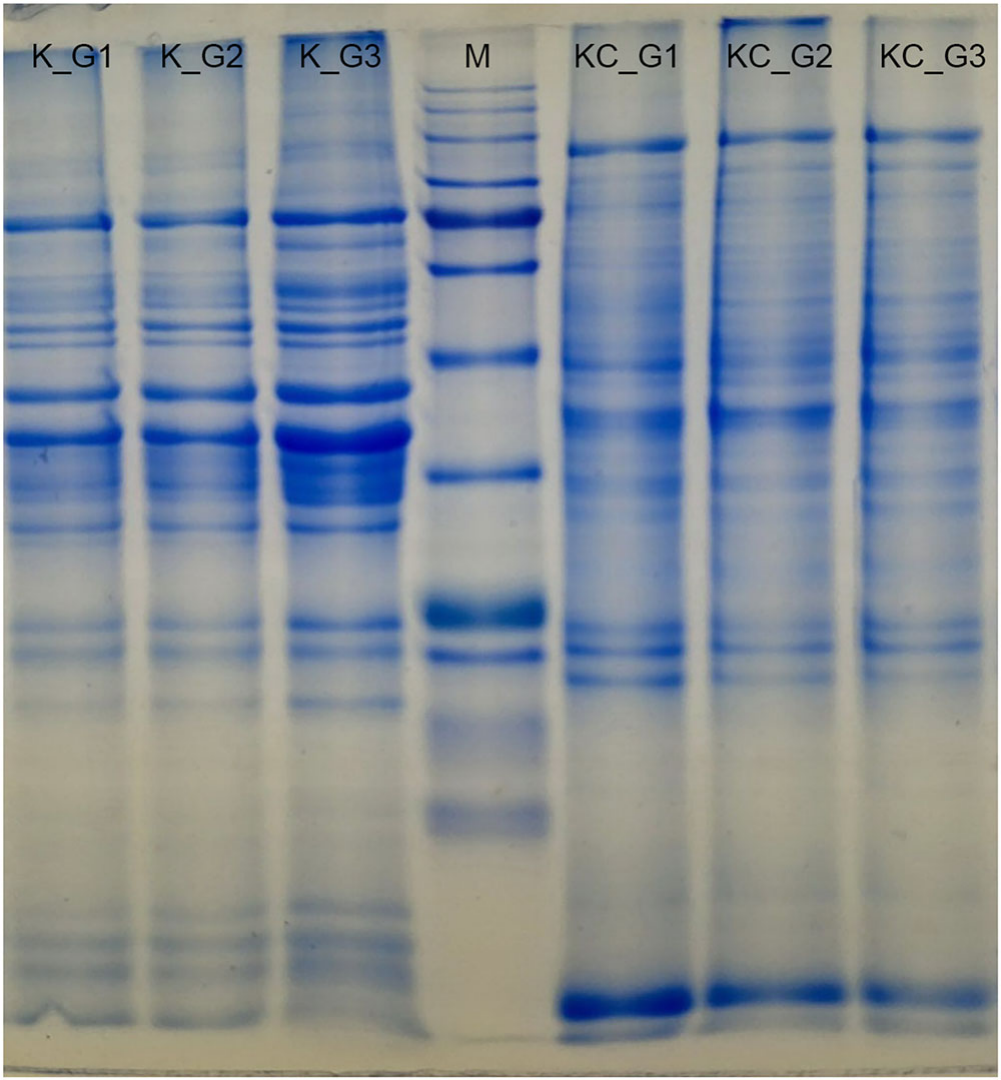
Protein profiles (M: Marker) in muscle (K) and liver (KC) tissues of the experimental groups (G1: Control, G2: Diabetes, G3: Diabetes + Psyllium Husk Powder).

**FIGURE 5:**
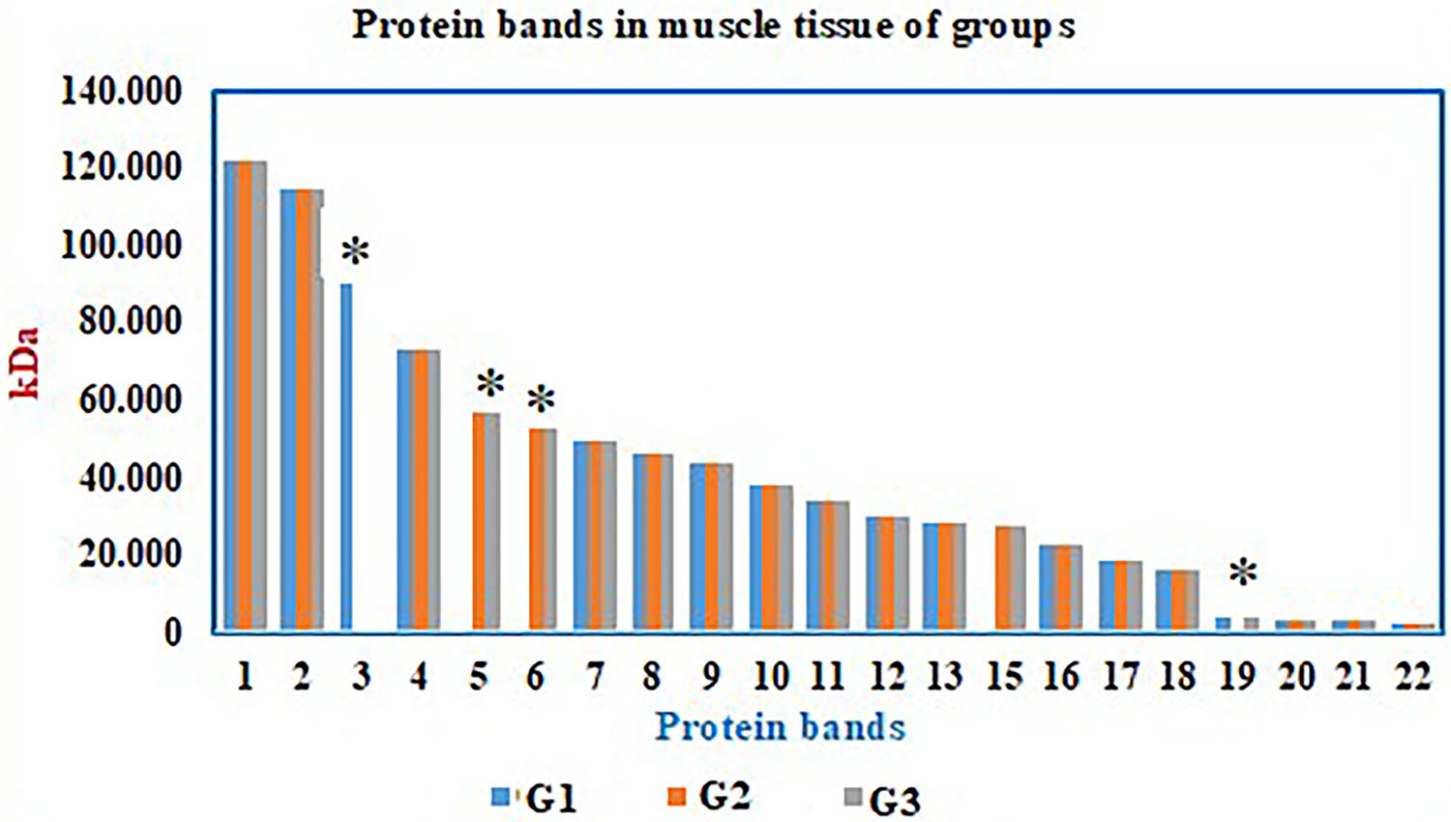
SDS‐PAGE protein bands in muscle tissue of groups (kDa). Protein bands from G1 (Control), G2 (Diabetic), and G3 (Diabetic + PHP) are shown. Significant differences between groups are indicated by asterisks (*p < 0.05). The 3rd protein band (89.66 kDa) was present only in the control group (G1) and absent in the diabetic groups (G2 and G3). The 5th (56.23 kDa) and 6th (52.75 kDa) protein bands were absent in the control group (G1) but present in the diabetic groups (G2 and G3). The 19th protein band (4.11 kDa) was detected only in the diabetic group receiving PHP (G3).

**FIGURE 6:**
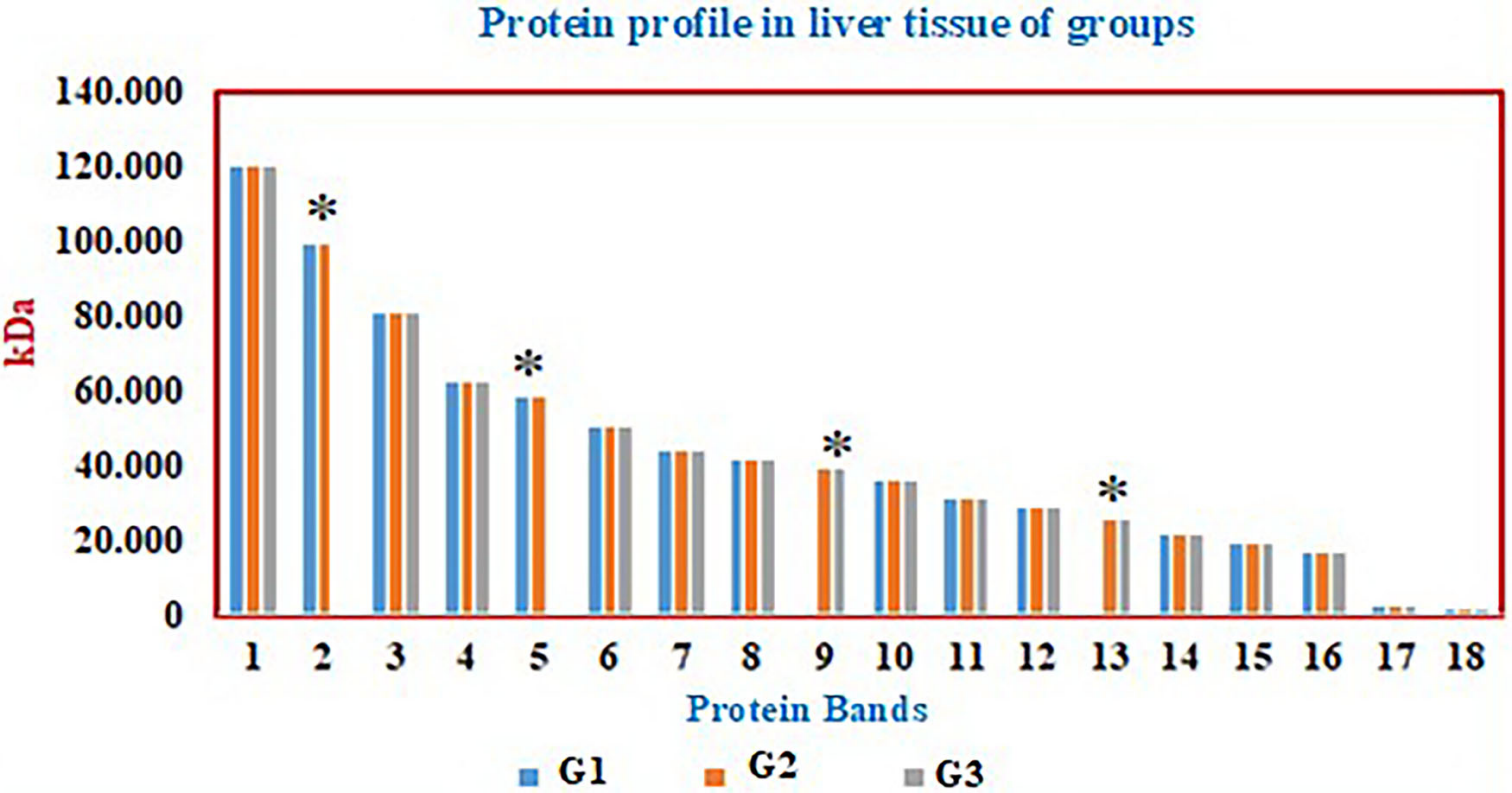
SDS‐PAGE protein bands in liver tissue of groups (kDa). Protein bands from G1 (Control), G2 (Diabetic), and G3 (Diabetic + PHP) are shown. Significant differences between groups are indicated by asterisks (*p < 0.05). In the control group (G1), 16 protein bands were identified, while G2 (Diabetic) displayed 18 protein bands, and G3 (Diabetic + PHP) exhibited 16 bands. The 2nd (98.83 kDa) and 5th (58.45 kDa) protein bands were present in both the control (G1) and diabetic control (G2) groups but absent in the diabetic group treated with PHP (G3). The 9th (39.31 kDa) and 13th (25.83 kDa) protein bands were absent in the control group but present in the diabetic (G2) and PHP‐treated (G3) groups.

Protein profiles of muscle and liver tissue homogenates from experimental groups Group 1, Group 2 and Group 3 were analysed using the SDS‐PAGE method (Figures 4, 5, 6).

Protein profiles in muscle and liver tissues were analysed across three groups. In muscle tissue, 18 protein bands were detected in the control group (Group 1), while the diabetes group (Group 2) showed an increased protein expression with 20 bands, and 21 protein bands were identified in the diabetes + PHP group (Group 3). Notably, the 89.6 kDa band was expressed only in the control group and was not expressed in either of the diabetic groups. The bands at 56.2 kDa and 52.7 kDa were expressed exclusively in the diabetic groups and were not detected in the control group. Other bands, such as those at 121.6 kDa, 114.2 kDa, and 72.6 kDa, were found to be consistently expressed across all groups.

In liver tissue, 16 protein bands were detected in the control group, 18 bands in the diabetes group, and 16 bands in the treated group. The bands at 98.8 kDa and 58.4 kDa were expressed in both the control and diabetes groups, but were not observed in the treated group. Conversely, the bands at 39.3 kDa and 25.8 kDa were not expressed in the control group but were expressed in both the diabetic and treated groups. Other protein bands were found to be expressed in all groups. These findings demonstrate that diabetes alters the expression of specific protein bands in both muscle and liver tissues, with certain proteins being either expressed or not expressed depending on the disease state. PHP treatment modulated these expression patterns, indicating its potential influence on protein expression related to diabetes pathology and its mitigation.

**FIGURE 7:**
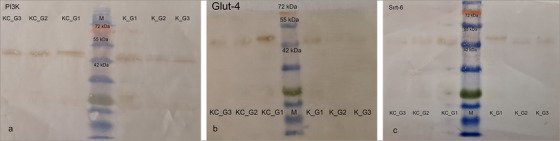
PI3K (a) Glut‐4 (b) and SIRT6 (c) protein expression in muscle and liver tissue of groups.

Glut‐4 protein expression in muscle tissue was highest in the control group (Group 1) and showed a modest increase in the diabetes + PHP group (Group 3) compared to the diabetic group (Group 2). No significant differences were observed in the expression levels of PI3K and SIRT6 proteins among the groups in muscle tissue (Figure 7).

In muscle and liver tissues, the expression levels of Glut‐4, PI3K, and SIRT6 proteins were observed to be higher in the control group (Group 1) compared to the diabetic groups, whereas reduced expression levels of these proteins were noted in the diabetic groups. Although psyllium husk administration showed a stimulatory effect on the expression of Glut‐4, PI3K, and SIRT6 proteins, this increase did not reach the levels observed in the control group.

## 4 Discussion

T2DM is a metabolic disease characterised by insulin resistance, hyperglycaemia, and/or insufficient insulin secretion. Due to its complex and not yet fully understood pathogenesis, research in this area is actively ongoing. This study aimed to investigate the effects of PHP on metabolic (SIRT6), hormonal (insulin, C‐peptide, IGF‐1), and appetite‐related (Nesfatin‐1, GLP‐1, Leptin) parameters, as well as antioxidant/oxidant balance and insulin signalling proteins (GLUT4, PI3K, IRS‐1, IRS‐2) in experimental T2DM rats.

An experimental T2DM model was induced by feeding rats a high‐fat diet for four weeks followed by a low‐dose STZ injection (Furman 2021; Sasidharan et al. 2013). Afterward, rats with induced T2DM were given PHP mixed with a 10% high‐fat diet and administered to Group 3. Native to regions such as Iran and India (Wei et al. 2009), psyllium has demonstrated therapeutic potential in managing metabolic and gastrointestinal disorders, including diabetes. Prior studies have shown that its consumption reduces blood glucose levels (Darooghegi Mofrad et al. 2020; Giuntini et al. 2022).

While high dietary fibre intake is generally associated with reduced body weight and improved metabolic health, the effects of psyllium supplementation have shown inconsistent results. For instance, while some studies suggest that psyllium supplementation may aid in weight management by enhancing satiety and reducing energy intake (Anderson et al. 1999), others have reported no significant effect on body weight, body mass index (BMI), or waist circumference in adults (Darooghegi Mofrad et al. 2020; Pal et al. 2011). Although no significant differences in body weight were observed between the groups at the beginning of the study, the greatest weight gain was recorded in the control group (G1) by the end of the experiment (week 16). This finding was consistent with the expected physiological weight gain in healthy animals fed a standard diet without metabolic impairment. In contrast, weight loss was detected in the diabetic groups (G2 and G3), which aligns with previous reports of body weight reduction in STZ‐induced diabetes models (Furman 2021). STZ induces damage to pancreatic β‐cells, leading to decreased insulin secretion, impaired glucose uptake, and ultimately the loss of fat and muscle tissue due to energy deficiency (Szkudelski 2001). In Group 3, although diabetic rats were supplemented with 10% psyllium husk powder (PHP for 12 weeks, only a slight decrease in body weight was observed compared to Group 2, and this difference was not statistically significant (*p* > 0.05). Psyllium, due to its soluble fibre content, has been reported to delay gastric emptying, enhance satiety, and improve insulin sensitivity by slowing glucose absorption (Anderson et al. 1999). Indeed, several studies have shown that psyllium supplementation may aid in weight management and glycaemic regulation in diabetic models (Ogata et al. 2017; Sierra et al. 2002). However, in the present study, the effect on body weight was limited, which may be attributed to the dosage, duration of intervention, or both.

In the present study, diabetes was successfully induced in rats using a HFD followed by a single intraperitoneal injection of STZ. This was confirmed by persistent hyperglycaemia (>200 mg/dL), elevated insulin levels, and significant changes in HOMA‐IR and HOMA‐β indices. These findings are consistent with previous reports indicating that HFD/STZ models closely replicate the pathophysiology of type 2 diabetes, including insulin resistance and β‐cell dysfunction (Furman 2021; Srinivasan and Ramarao 2007). The strong positive correlation observed between glucose levels and HOMA‐IR (*r* = 0.974**), and the negative correlation between HOMA‐IR and HOMA‐β (*r* = −0.674**), further support the presence of metabolic disturbances and impaired β‐cell function, which are characteristic features of insulin resistance in this model (Matthews et al. 1985; Wallace et al. 2004). Interestingly, although both diabetic groups exhibited marked metabolic impairments compared to the control group, supplementation with PHP in Group 3 resulted in significant reductions in blood glucose levels at several time points and improvements in insulin sensitivity markers (*p* < 0.05). While these improvements did not fully normalise the parameters to control levels, they suggest a beneficial role of psyllium in glycaemic regulation. Psyllium, a soluble fibre, is known to form a viscous gel in the gut, thereby delaying carbohydrate absorption and reducing postprandial glucose spikes (Anderson et al. 1999). Recent studies also support its regulatory effects on insulin sensitivity and β‐cell function, particularly in diabetic models (Ogata et al. 2017; Pal et al. 2011). Furthermore, the reduction in HOMA‐IR and partial preservation of HOMA‐β observed in the PHP‐supplemented group may reflect improved insulin signalling and reduced glucotoxicity, potentially mediated through short‐chain fatty acid (SCFA) production and modulation of the gut microbiota (Slavin 2013; Zhao et al. 2018). Nevertheless, the fact that metabolic parameters in Group 3 remained significantly different from those of the control group suggests that psyllium supplementation alone may not be sufficient to fully reverse diabetic alterations, particularly under continued dietary fat overload. Future studies should explore the synergistic effects of psyllium in combination with pharmacological agents or other dietary interventions to more effectively reverse insulin resistance and pancreatic dysfunction.

In this study, streptozotocin‐induced diabetes in rats resulted in significant alterations in serum, liver, and muscle biochemical profiles, reflecting systemic metabolic dysregulation. Notably, serum levels of TC, TG, and CRE were significantly elevated in the diabetic group. These findings are consistent with previous reports indicating that insulin deficiency or resistance disrupts lipid metabolism and promotes renal dysfunction (Gheith et al. 2015; Giacco and Brownlee 2010). In Group 3, supplementation with PHP led to significant reductions in TC, TG, and CRE levels compared to the untreated diabetic group, with values approaching those of the control group. These results suggest a potential lipid‐lowering and renoprotective effect of psyllium, likely due to its soluble, viscosity‐forming fibre content, which can bind bile acids and reduce cholesterol absorption (Anderson et al. 1999; Pal et al. 2011). Although serum levels of TP, ALB, U, and UA did not differ significantly among the groups, tissue‐specific changes were observed. In muscle tissue, urea and triglyceride levels were significantly elevated in diabetic rats, while PHP supplementation partially attenuated these increases. Interestingly, total protein (K‐TP) levels were significantly higher in the PHP‐supplemented group compared to controls, suggesting enhanced protein synthesis or retention in muscle tissue under the influence of psyllium. Similar tissue‐protective effects of psyllium have previously been reported in diabetic animal models and may be associated with its anti‐inflammatory and gut‐regulatory properties (Slavin 2013; Zhao et al. 2018). Moreover, glucose levels in both liver (KC‐Glu) and muscle (K‐Glu) tissues were significantly elevated in the diabetic and PHP‐supplemented groups, reflecting chronic hyperglycaemia induced by STZ. However, glucose levels were lower in Group 3 compared to Group 2, suggesting that PHP may help alleviate tissue‐level glucotoxicity. In parallel, decreased uric acid levels in liver and muscle tissues of diabetic animals are likely indicative of altered purine metabolism and oxidative stress, as reported in previous diabetes‐related studies (Dehghan et al. 2008). The partial normalisation of these parameters with psyllium supplementation points to a possible antioxidant or anti‐inflammatory role, though further research is needed to clarify the mechanisms involved. Correlation analysis supported these observations by revealing several significant associations among tissue parameters. The strong positive correlations between KC‐Glu and K‐TG (*r* = 0.530**) and between K‐Glu and K‐URE (*r* = 0.586**) suggest that hyperglycaemia in diabetes may be associated with increased triglyceride accumulation and elevated nitrogen metabolism in tissues. The negative correlation between K‐TP and KC‐TG (*r* = −0.372*) supports the idea that muscle protein status may be adversely affected by hepatic lipid accumulation. Additionally, the positive correlation between KC‐TK and KC‐TP (*r* = 0.897**) may reflect coordinated changes in hepatic enzyme activity and protein metabolism under diabetic conditions. Overall, these findings demonstrate that diabetes induces multi‐tissue metabolic impairments, and while psyllium husk supplementation does not fully reverse these alterations, it provides partial improvements in lipid, glucose, and renal parameters. However, the lack of significant changes in some markers (TP, ALB, UA) and the persistence of certain diabetic alterations suggest that the therapeutic potential of psyllium may be limited when used alone, particularly under continuous HFD conditions. Future studies should explore its use in combination with other dietary or pharmacological strategies to enhance metabolic regulation.

In this study, significant changes in oxidative stress markers were observed among the groups, as indicated by serum levels of TAS, TOS, and OSI (Table 6). The diabetic group (G2) showed significantly elevated TOS and OSI levels and a marked reduction in TAS levels compared to the control group (G1), reflecting an imbalance in redox homeostasis. These findings are consistent with previous reports indicating that diabetes is associated with increased production of reactive oxygen species (ROS) and impaired antioxidant defence mechanisms (Maritim et al. 2003). Chronic hyperglycaemia promotes oxidative stress through multiple pathways, including glucose auto‐oxidation, polyol pathway activation, advanced glycation end‐product (AGE) formation, and mitochondrial dysfunction (Brownlee 2005). In the psyllium‐supplemented group (G3), TAS levels were significantly higher and OSI values were significantly lower than in the untreated diabetic group (G2), though not fully restored to control levels. This partial restoration suggests that psyllium husk powder may exert a protective effect against oxidative stress, likely due to its high soluble fibre content and its potential to modulate gut microbiota, reduce postprandial glucose spikes, and decrease systemic inflammation (Anderson et al. 1999; Slavin 2013; Zhao et al. 2018). Furthermore, dietary fibres such as psyllium have been shown to indirectly enhance antioxidant capacity by promoting the production of short‐chain fatty acids (SCFAs), particularly butyrate, which can upregulate the expression of antioxidant enzymes (Zhou et al. 2020). The correlations observed in Table 8 further support these interactions. A strong positive correlation was detected between TAS and OSI (*r* = 0.893*, *p* < 0.01), indicating that changes in antioxidant capacity closely reflect overall oxidative status. Meanwhile, a moderate negative correlation between TOS and OSI (*r* = –0.548*, *p* < 0.01) suggests that as total oxidant levels increase, oxidative stress burden also rises proportionally. Although the inverse correlation between TAS and TOS (*r* = –0.196) was not statistically significant, it aligns with the conceptual framework of oxidative stress imbalance in diabetes (Giugliano et al. 1996). Overall, these findings confirm that oxidative stress plays a key role in the pathogenesis of diabetes and its complications. Psyllium husk supplementation, although not completely reversing oxidative damage, may offer partial protection by enhancing antioxidant defences and reducing oxidant burden. However, the persistence of elevated TOS levels despite improvements in TAS and OSI suggests that future studies should investigate combination therapies or higher doses and longer durations of fibre supplementation.

In this study, SDS‐PAGE analysis revealed significant changes in the protein expression profiles of muscle and liver tissues in diabetic rats, and demonstrated that PHP supplementation modulated these expression patterns. The diabetic condition caused an increase or decrease in the expression of certain protein bands associated with metabolic stress and impaired glucose regulation. In muscle tissue, the loss of the 89.6 kDa protein band and the emergence of bands at 56.2 kDa and 52.7 kDa exclusively in the diabetic groups indicated diabetes‐induced proteomic remodelling. These changes may be linked to increased oxidative stress, protein degradation, and altered muscle metabolism associated with insulin resistance (Li et al. 2013). The increase in the total number of protein bands in the PHP‐supplemented group suggests a partial enhancement of protein synthesis or stabilisation, reflecting the beneficial effects of PHP on diabetic muscle catabolism. Similarly, alterations in protein expression associated with diabetes were observed in liver tissue. Some protein bands (39.3 kDa and 25.8 kDa) exhibited increased expression in both the diabetic and treated groups, whereas others (98.838 kDa and 58.4 kDa) were absent in the treated group. This suggests that hyperglycaemia and associated metabolic disturbances induce specific changes in hepatic protein expression. PHP supplementation appears to contribute to the regulation of proteostasis by partially normalising these expression profiles in the liver. When examining the expression of Glut‐4, PI3K, and SIRT6 proteins, Glut‐4 expression was highest in the control group, significantly reduced in the diabetic group, and partially restored with PHP treatment. Given Glut‐4's role as the insulin‐regulated glucose transporter in muscle and adipose tissues, its reduction is a commonly reported feature in diabetes (Huang and Czech 2007). The partial restoration observed with PHP may be related to the modulation of gut hormones and systemic inflammation by dietary fibre, leading to improved insulin sensitivity and glucose uptake (Anderson et al. 1999; Zhao et al. 2018). Although no statistically significant differences were observed in PI3K and SIRT6 expression among groups, a trend of decreased expression in diabetic groups and partial recovery in the PHP group was noted. PI3K is a critical mediator of insulin signalling facilitating Glut‐4 translocation, while SIRT6 regulates glucose metabolism, mitochondrial function, and inflammatory responses. Reduced SIRT6 expression in diabetes has been linked to metabolic dysfunction and tissue aging (Xiao et al. 2010). The ability of PHP to enhance the expression of these proteins may be connected to its known effects in reducing oxidative stress and improving insulin sensitivity.

## 5 Conclusion

In this study, supplementation with PHP in a streptozotocin‐induced type 2 diabetes model was found to provide partial improvements in metabolic parameters, oxidative stress, and protein expression profiles. PHP was shown to mitigate the adverse effects of diabetes by reducing blood glucose levels, enhancing insulin sensitivity, and supporting protein synthesis in muscle and liver tissues. However, since PHP did not fully reverse all pathophysiological disturbances associated with diabetes, further studies involving combination therapy strategies and longer‐term interventions are recommended. In conclusion, psyllium husk powder was identified as a potential complementary approach in the management of type 2 diabetes.

## Author Contributions

Gülay Çiftci (corresponding author) supervised the project, performed the statistical analysis, interpreted the results, organised the tables and figures, and wrote the paper. Burcu Onuk, Furkan Ümit, and Özüm Çaka conducted the experiments, designed the study, and supported the research. Furkan Ümit and Recai Aci prepared the samples and performed the analyses. Alper Çiftci performed the tape analyses of the tapes. All authors read and approved the final draft.

## Funding

This research was supported by the Scientific Research Projects Commission of Ondokuz Mayis University with the Contract Grand Number of PYO.VET.1901.23.001.

## Ethics Statement

The research was approved by the Ondokuz Mayıs University Animal Experiments Local Ethics Committee (HAYDEK) with Ethics Committee Decision No. 2022/58.

## Conflicts of Interest

The authors declare no competing interests.

## Data Availability

The data that support the findings of this study are available on request from the corresponding author. The data are not publicly available due to privacy or ethical restrictions.
